# Insulin enhancement of the antitumor activity of chemotherapeutic agents in colorectal cancer is linked with downregulating PIK3CA and GRB2

**DOI:** 10.1038/s41598-019-53145-x

**Published:** 2019-11-12

**Authors:** Siddarth Agrawal, Marta Woźniak, Mateusz Łuc, Sebastian Makuch, Ewa Pielka, Anil Kumar Agrawal, Joanna Wietrzyk, Joanna Banach, Andrzej Gamian, Monika Pizon, Piotr Ziółkowski

**Affiliations:** 1 Department of Pathology, Wroclaw Medical University, Wroclaw, Poland; 2 2nd Department and Clinic of General and Oncological Surgery, Wroclaw Medical University, Wroclaw, Poland; 3 Department of Experimental Oncology, Ludwik Hirszfeld Institute of Immunology and Experimental Therapy, Polish Academy of Sciences, Wroclaw, Poland; 4 Department of Biochemistry, Wroclaw Medical University, Wroclaw, Poland; 5 Transfusion Center Bayreuth, Kurpromenade 2, 95448 Bayreuth, Germany

**Keywords:** Chemotherapy, Cancer metabolism

## Abstract

The present state of cancer chemotherapy is unsatisfactory. New anticancer drugs that marginally improve the survival of patients continue to be developed at an unsustainably high cost. The study aimed to elucidate the effects of insulin (INS), an inexpensive drug with a convincing safety profile, on the susceptibility of colon cancer to chemotherapeutic agents: 5-fluorouracil (FU), oxaliplatin (OXA), irinotecan (IRI), cyclophosphamide (CPA) and docetaxel (DOC). To examine the effects of insulin on cell viability and apoptosis, we performed an *in vitro* analysis on colon cancer cell lines Caco-2 and SW480. To verify the results, we performed *in vivo* analysis on mice bearing MC38 colon tumors. To assess the underlying mechanism of the therapy, we examined the mRNA expression of pathways related to the signaling downstream of insulin receptors (INSR). Moreover, we performed Western blotting to confirm expression patterns derived from the genetic analysis. For the quantification of circulating tumor cells in the peripheral blood, we used the maintrac method. The results of our study show that insulin-pretreated colon cancer cells are significantly more susceptible to commonly used chemotherapeutics. The apoptosis ratio was also enhanced when INS was administered complementary to the examined drugs. The *in vivo* study showed that the combination of INS and FU resulted in significant inhibition of tumor growth and reduction of the number of circulating tumor cells. This combination caused a significant downregulation of the key signaling substrates downstream of INSR. The results indicate that the downregulation of PIK3CA (phosphatidylinositol 3-kinase catalytic subunit alpha), which plays a critical role in cell signaling and GRB2 (growth factor receptor-bound protein 2), a regulator of cell proliferation and differentiation may be responsible for the sensitizing effect of INS. These findings were confirmed at protein levels by Western blotting. In conclusion, these results suggest that INS might be potentially applied to clinical use to enhance the therapeutic effectiveness of chemotherapeutic drugs. The findings may become a platform for the future development of new and inexpensive strategies for the clinical chemotherapy of tumors.

## Introduction

Colorectal cancer (CRC) is one of the most frequent malignancies and one of the leading causes of cancer-related deaths in the world^1^. The treatment outcome of patients diagnosed with unresectable CRC is poor; it is estimated that only one out of two patients will respond to the classical chemotherapy^2,3^. The present state of systemic cancer therapy is unsatisfactory, as new anticancer drugs continue to be developed and approved by marginal improvements in survival at an unsustainably high financial cost^4^. As a result, it would seem more rational to attempt to improve the treatment outcome by implementing inexpensive treatments with a convincing safety profile.

Classical chemotherapy, which exerts its anticancer action by causing damage and inducing programmed cell death, particularly in rapidly growing tumors with high-growth fractions cells, has been a foundation in standard cancer treatment for many years. The rationale for using standard chemotherapy is to kill cancer cells in order to reduce tumor size. However, many solid tumors including CRC are slowly growing malignancies and have relatively more cells with a low-growth fraction and therefore are less susceptible to chemotherapy^5^.

It has been reported that insulin as a pharmacological agent induces the switch from a noncycling to a cycling status and therefore strongly modifies the metabolism of malignant cells^6,7^. Moreover, as a classic hormone, it affects lipid synthesis, carbohydrate metabolism, cell proliferation, motility and survival^8^. Several *in vitro*^9–14^ and clinical studies^15,16^ implicated insulin pretreatment may play a previously unknown significant role in increased drug uptake and cell susceptibility to cytotoxic therapy. Therefore, this study was carried out to validate the insulin-induced enhancement of the antitumor effect of widely incorporated cytotoxic agents 5-fluorouracil (FU), oxaliplatin (OXA), irinotecan (IRI), cyclophosphamide (CPA) and docetaxel (DOC). Moreover, to establish the mechanisms underlying this phenomenon, we assessed the mRNA expression of pathways related to the signaling downstream of insulin receptors.

## Results

### Insulin pretreatment enhances cytotoxicity

**Figure 1:**
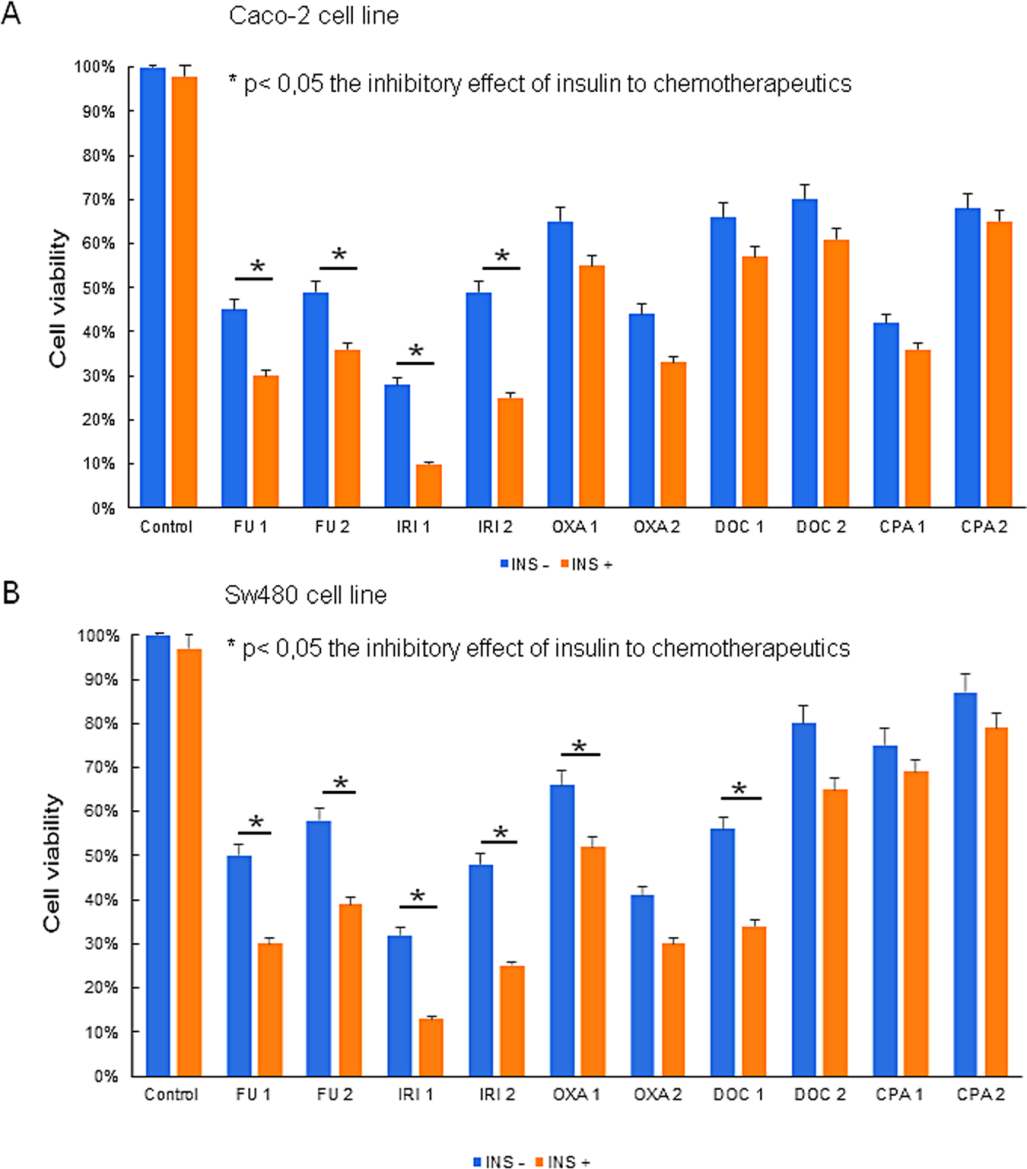
After an 8-hour insulin (INS) pretreatment (100 µg/ml) Caco-2 cells (**A**) were exposed to 5-fluorouracil (FU) (1) 500 µM, (2) 100 µM; irinotecan (IRI) (1) 150 µM, (2) 50 µM; oxaliplatin (OXA) (1) 50 µM, (2) 15 µM; docetaxel (DOC) (1) 4000 nM, (2) 1000 nM; cyclophosphamide (CPA) (1) 15 µM, (2) 4 µM and SW480 cells (**B**) were treated with FU (1) 500 µM, (2) 250 µM; IRI (1) 200 µM, (2) 100 µM; OXA (1) 198 µM, (2) 96 µM; DOC (1) 4000 nM, (2) 100 nM; CPA (1) 12 µM, (2) 6 µM for 48 hours. The inhibitory effect was measured by MTT assay. The results are shown as mean ± SD from three individual experiments. Statistically significant variables were marked with *(p < 0.05).

The MTT viability assay showed that colon cancer cells sensitized by insulin are more susceptible to chemotherapeutic drugs (Fig. 1). We found that a combination of FU with insulin led to a significant decrease in viability in Caco-2 and SW480 colon cancer cells compared with FU alone. The effect was observed both in higher (500 µM) and a lower (100 µM) concentration of FU. A similar effect was reported in IRI-treated cells. The prior administration of insulin resulted in a significant enhancement of the cytotoxic effect of the drug. We found a two-fold decrease in the viability of cells pretreated with insulin. Interestingly, we found that OXA and DOC (in higher concentration 198 µM and 4000 µM, respectively) displayed a significantly enhanced cytotoxicity in the presence of insulin only in SW480 cells, which have a higher metastatic potential than Caco-2 cells^17^.

### Insulin and FU/IRI increase apoptosis

**Figure 2:**
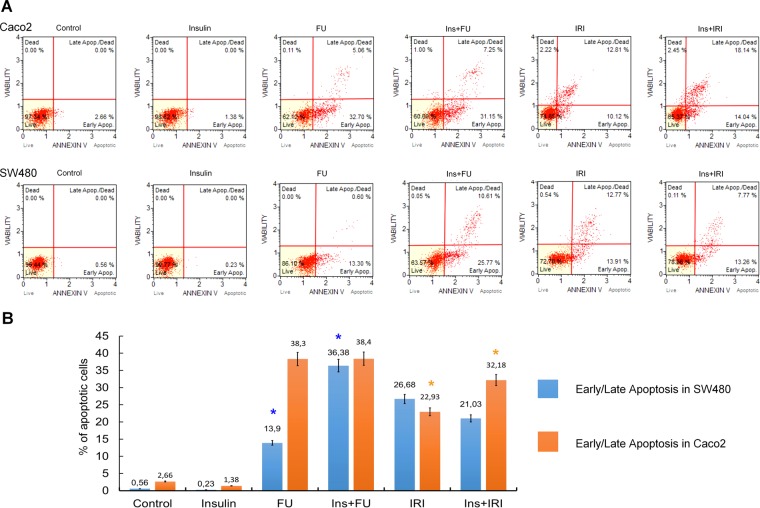
Effects of 5-fluorouracil (FU) and irinotecan (IRI) with and without additional insulin (INS) pretreatment on apoptosis of Caco-2 and SW480 cancer cells. Caco-2 and SW480 carcinoma cells were treated with 5-fluorouracil (FU) and irinotecan (IRI) in the concentration of 500 µM and IRI 50 µM, respectively for 48 h. (**A**) Original histogram plots include a percentage of live, early apoptotic, late apoptotic, total apoptotic, and dead cells differentiated using Muse® Annexin V and Dead Cell Assay Kit. (**B**) The graph bar presents a statistical analysis of the early/late apoptosis in different samples. The results are shown as mean ± SD from two individual experiments. Asterisks indicate significant differences between the groups (FU vs. Ins + FU in SW480 and IRI vs. Ins + IRI in Caco-2; p < 0.05).

The flow cytometry analysis revealed a greater percentage of apoptosis after treating cells with chemotherapeutics (Fig. 2). This effect was enhanced 2-fold when SW480 cells were also pretreated with insulin before using FU. Caco-2 cells exhibited up to 50% greater apoptotic percentage when IRI treated cells were additionally pretreated with insulin. These results indicate that even though the effect can vary depending on tested cell lines and drugs, the influence of insulin on colon cancer cells remain.

### Insulin and FU/IRI alter the cellular metabolism through regulating the mRNA expression of PIK3-related genes and proteins

**Figure 3:**
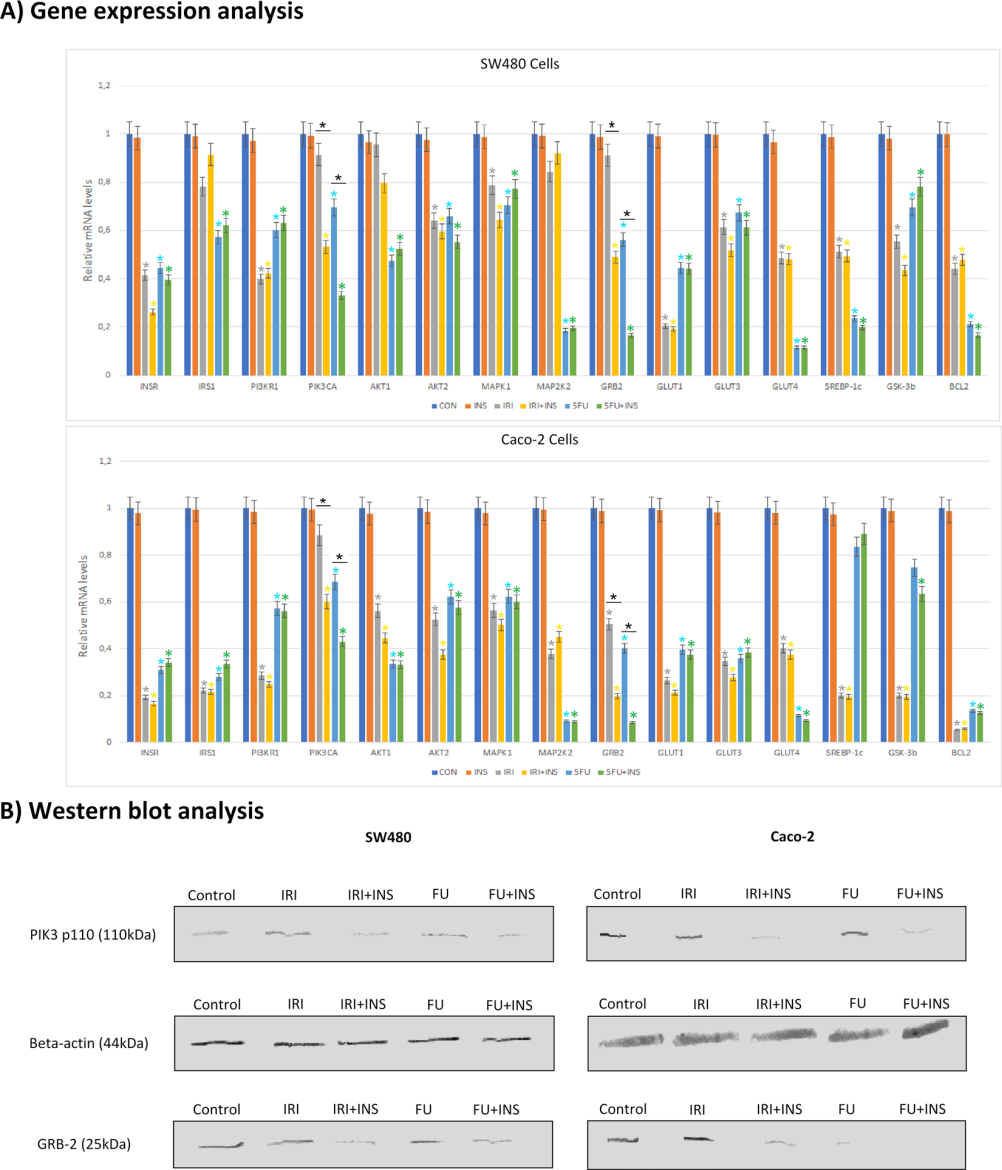
(**A**) Gene expression analysis of substrates involved in the pathways related to the signaling downstream of insulin (INS) receptors by quantitative RT-PCR. Caco-2 and SW480 carcinoma cells were treated with 5-fluorouracil (FU) and irinotecan (IRI) in the concentration of 500 µM and IRI 50 µM, respectively for 48 h. The mRNA expression of INSR (insulin receptor), IRS 1 (insulin receptor substrate 1), PIK3CA (phosphatidylinositol 3-kinase catalytic subunit alpha), PIK3R1 (phosphatidylinositol 3-kinase regulatory subunit alpha), AKT1 and AKT2 (AKT Serine/Threonine Kinase 1 and 2), MAPK1 (Mitogen-Activated Protein Kinase 1), MAP2K2 (Mitogen-Activated Protein Kinase Kinase 2), GRB2 (Growth factor receptor-bound protein 2), glucose transporters (GLUT-1, GLUT-3, GLUT-4), SREBP-1c (sterol regulatory element-binding protein-1c), GSK3B (Glycogen synthase kinase 3 beta), antiapoptotic protein BCL-2, caspase 3 (CASP3) was determined by quantitative RT-PCR using gene-specific primers. Data are presented as mean ± SD. Experiments were run in triplicate and carried out once. *P < 0.05 compared with control group. (**B**) Western blotting analysis of expression of PIK3CA and GRB2 in Caco-2 and SW480 cancer cell lines.

To examine the effect of the therapy on cell metabolism, we examined the mRNA expression of pathways related to the signaling downstream of insulin receptors. We have found that FU and IRI caused significant downregulation of the key signaling substrates (Fig. 3A). The administration of drugs to Caco-2 colon cancer cells and SW480 cells resulted in a significant inhibition of INSR (insulin receptor), IRS 1 (insulin receptor substrate 1), PIK3R1 (phosphatidylinositol 3-kinase regulatory subunit alpha), AKT1 and AKT2 (AKT Serine/Threonine Kinase 1 and 2), MAPK1 (Mitogen-Activated Protein Kinase 1), MAP2K2 (Mitogen-Activated Protein Kinase Kinase 2), SREBP-1c (sterol regulatory element-binding protein-1c) and GSK3B (Glycogen synthase kinase 3 beta). Moreover, we have found significant downregulation of glucose transporters (GLUT-1, GLUT-3, GLUT-4) and anti-apoptotic protein BCL-2. Interestingly, additional pretreatment with insulin resulted in a significantly lower expression of PIK3CA (phosphatidylinositol 3-kinase catalytic subunit alpha), which plays a critical role in cell signaling and GRB2 (growth factor receptor-bound protein 2), a regulator of cell proliferation and differentiation. These findings were confirmed for both cell lines at protein levels by Western blotting (Fig. 3B).

### *In vivo* activity against colon cancer tumors

**Figure 4:**
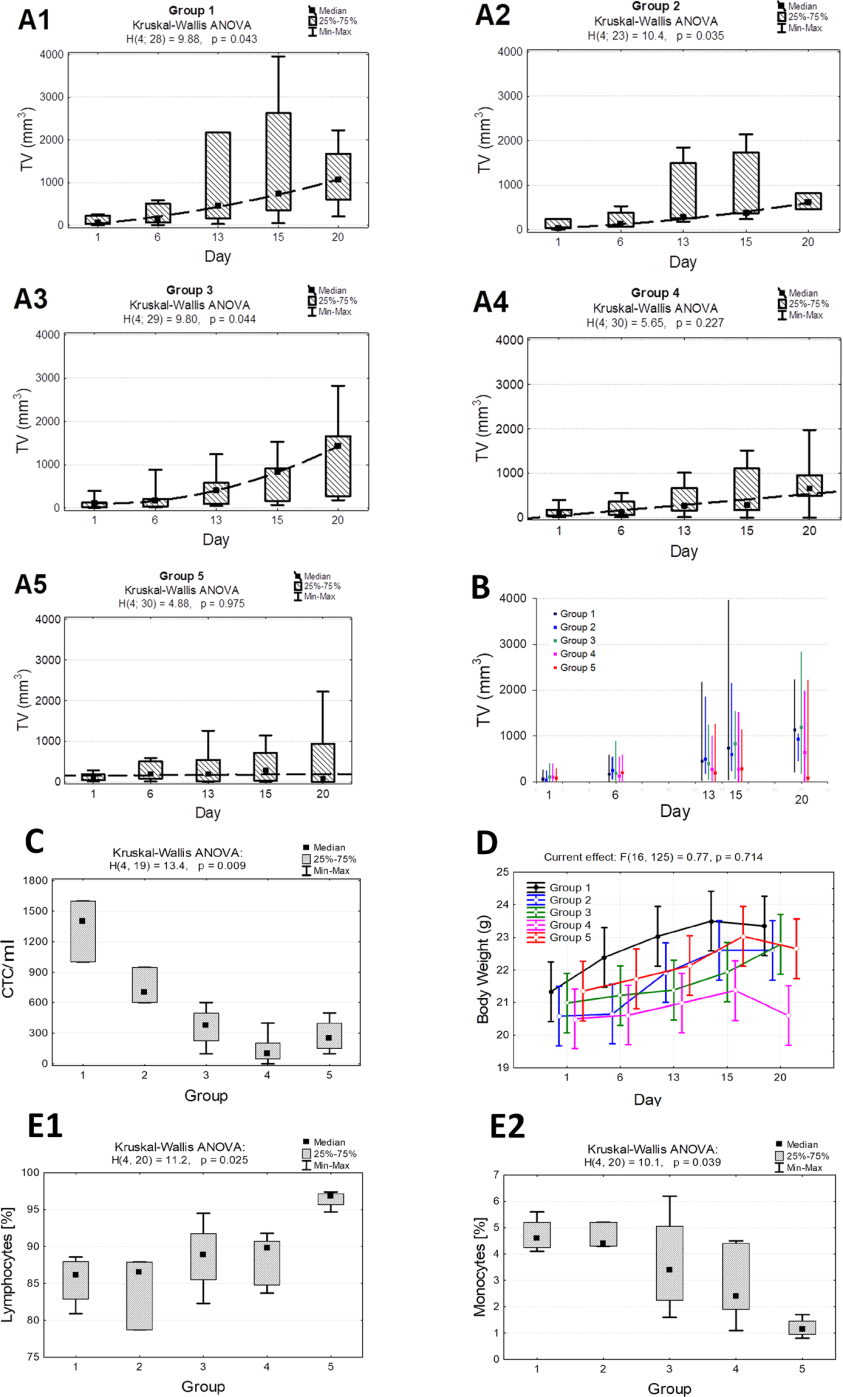
Results of the *in vivo* study. The animals bearing mouse colon tumor (MC38 cell line) were divided into five groups (10 mice per group): 1- control, 2- insulin (INS) only, 3- FU only, 4- insulin with FU administered together, 5- insulin administered 60 minutes before FU. (**A**) Non-parametric Kruskal-Wallis ANOVA analysis of the tumor volume in the following days of the experiment. Presented three groups (control, insulin, FU) show statistically significant tumor increase in comparison to groups 4 and 5 (combination insulin and FU) where slower tumor growth was observed. (**B**) The average tumor volume after the 3-weeks therapy in 5 groups. In group 5, the tumor volume was significantly lower compared to other groups (p = 0.037). (**C**) Kruskal-Wallis ANOVA for the comparison of the amount of the circulating epithelial tumor cells/ml of blood in 5 groups. Post-hoc comparisons for the CTCs analysis show that the number of CTC between control (group 1) and FU combined with insulin (group 4 and 5) was statistically significant (1,400 vs. 100; p = 0.013 and 1,400 vs. 250; p = 0.043, respectively). (**D**) The effect of the therapy on body weight in the following days of the experiment in mice differing in the treatment method (mean values and 95% confidence intervals). There was no significant loss of body weight in insulin and/or FU treated mice. (**E**) The percentage of lymphocytes was significantly lower in control animals (group 1) and those treated with insulin alone (group 2) compared with mice treated with FU combined with insulin (group 5) (86% vs. 97%; p = 0.041 and 87% vs. 97%; p = 0.045, respectively). The percentage of monocytes was also statistically significant between groups 1 and 5 (5% vs. 1%; p = 0.045).

**Figure 5:**
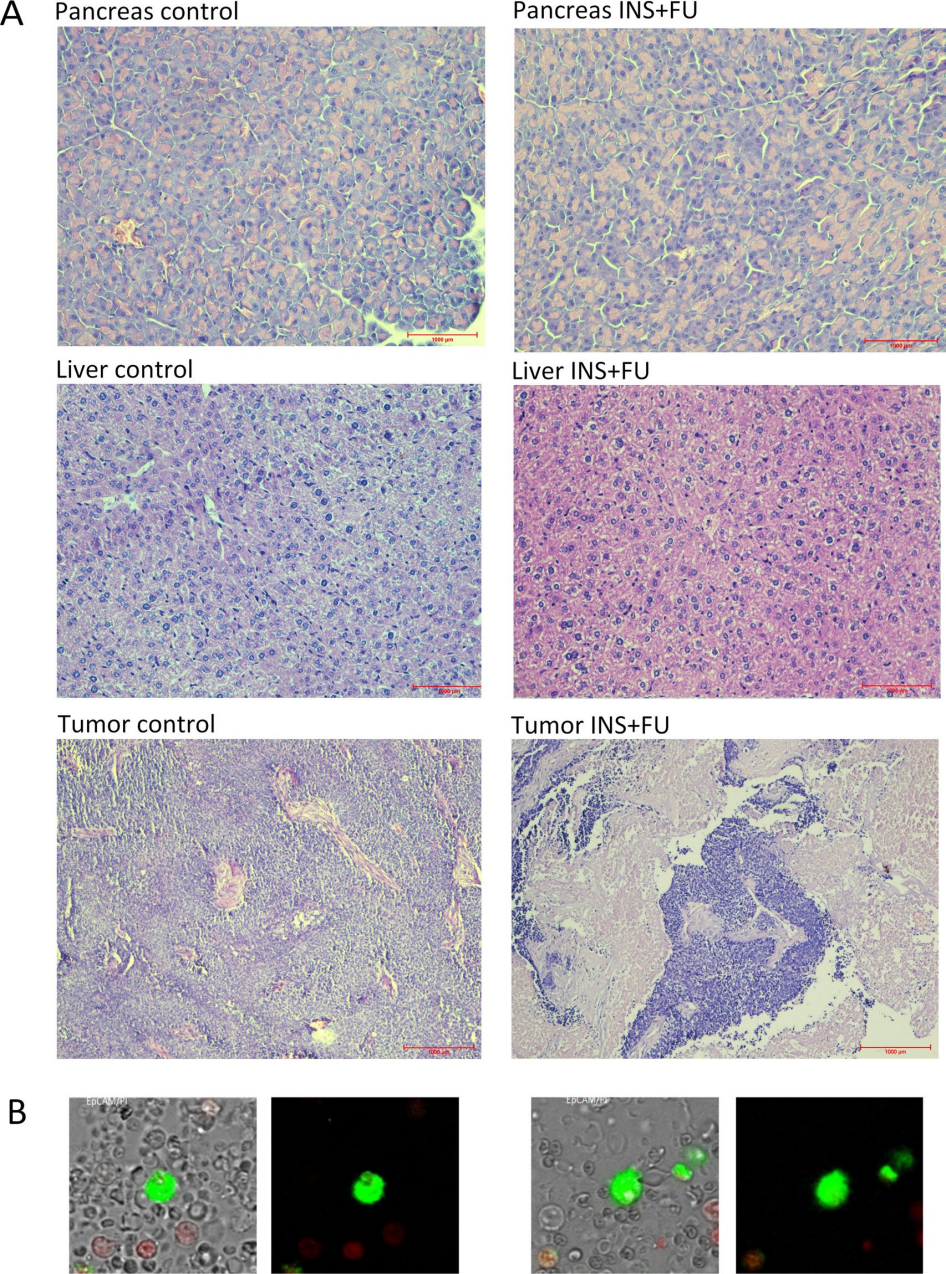
(**A**) Photographs of pancreas, liver, and tumor sections stained with hematoxylin-eosin (HE) to evaluate the impact of the insulin and chemotherapeutics on the tissues. The figure presents an organ from a control mouse and mouse treated with insulin and FU together (group 5). There are no significant changes in the morphology in the pancreas and liver. The figure of the tumor presents control, insulin, fluorouracil, and insulin together with fluorouracil (group 5) conditions. Sections of fluorouracil and fluorouracil with insulin reveals parts of the necrotic area in comparison to the control. Optical magnification: 200x (pancreas, liver) and 100x (tumor). (**B**) Illustrative images of CTCs detected in mouse blood. CTCs with a green EpCAM surface staining, a well-preserved morphology, but no red nuclear staining by PI is shown. The figure presents an image from a control mouse and mouse treated with insulin and FU together.

After our observations of *in vitro* effects, insulin and FU were evaluated in a mouse allograft model of colon cancer. There was a significant promotion in tumor weight in control as well as insulin and FU-only treated animals (p < 0.05 in groups 1 to 3, Table 1, and Fig. 4A). When the animals were treated or pretreated with insulin (groups 4 and 5, respectively) combined with FU, no raise in the tumor weight was observed. We found that the average tumor weight after the 3-weeks therapy was significantly lower in group 5 compared to other groups (p = 0.037, Fig. 4B). Insulin added to FU therapy was statistically different in the effects of FU alone (P < 0.05). Histopathological analyses of the tumors excised from control mice showed groups of large cells with different degrees of cellular and nuclear pleomorphism. Mitosis, muscle invasion, and coagulation necrosis were also noticed. In the tumors excised from treated animals, extensive areas of coagulative necrosis were observed (Fig. 5). Thus, the results indicate that insulin combined with FU significantly inhibited tumor growth when compared to FU or insulin alone. This indicated that insulin might increase the antitumor activity of FU. Interestingly, we found that there was a statistically significant difference in the number of CTC between control (group 1) and FU combined with insulin (groups 4 and 5) (1,400 vs. 100; p = 0.013 and 1,400 vs. 250; p = 0.043; Table 2 and Fig. 4C).

**Table 1:** Overview of the tumor volume measurement results (Med and Q1 and Q3) in the following days of the treatment in five groups of mice and the result of comparisons (non-parametric Kruskal-Wallis test).

Day	Groups of mice					*p*
	Group 1	Group 2	Group 3	Group 4	Group 5	
1	59 [32; 225]	40 [39; 237]	108 [17; 124]	106 [23; 192]	81 [27; 206]	0.997
6	166 [62; 512]	117 [70; 382]	182 [34; 211]	127 [26; 433]	200 [46; 581]	0.976
13	453 [163; 2169]	269 [257; 1494]	397 [95; 586]	268 [140; 768]	198 [0; 602]	0.738
15	737 [357; 2624]	376 [374; 1743]	834 [168; 911]	273 [150; 1358]	282 [0; 804]	0.515
20	1062 [602; 1679]	620 [461; 817]	1430 [278; 1662]	644 [493; 1006]	85 [0; 1182]	**0**.**037**
*p*	**0**.**043**	**0**.**035**	**0**.**044**	0.227	0.975	×

**Table 2:** Basic statistical analysis of the number of circulating tumor cells after therapy in five groups of mice differing in the treatment method and the result of comparisons (non-parametric Kruskal-Wallis test).

Parameter	Group					*p*
	Group 1	Group 2	Group 3	Group 4	Group 5	
CTC/μL						
*Me* ± *SD*	1333 ± 306	750 ± 180	363 ± 206	150 ± 158	275 ± 171	**0**.**009**
*Med* [*Q*1; *Q*3]	1400 [1000; 1600]	700 [600; 950]	375 [225; 500]	100 [50; 200]	250 [150; 400]	
*Min*–*Max*	1000–1600	600–950	100–600	0–400	100–500	

### Toxicological aspects of FU treatment with and without insulin

There was no significant loss of body weight in insulin and/or FU treated mice (Fig. 4D). Hematological parameters were measured after the experiment (Table 3). We found that the percentage of lymphocytes was significantly lower in control animals (group 1) and those treated with insulin alone (group 2), compared with mice treated with FU combined with insulin (group 5) (86% vs. 97%; p = 0.041 and 87% vs. 97%; p = 0.045, respectively) (Table 3 and Fig. 4E1). Moreover, we found that the percentage of monocytes was also statistically significant between groups 1 and 5 (5% vs. 1%; p = 0.045; Fig. 4E2). No other significant changes in hematological parameters were seen in any animals. The organs removed from the treated animals were weighed. No significant changes in the liver and pancreas were seen in any animals treated with insulin alone or in combination with FU or FU alone (Fig. 5).

**Table 3:** Basic statistical analysis of the hematologic test after the therapy in five groups of mice differing in the treatment method and the result of comparison (non- parametric Kruskal-Wallis test).

Parameter	Group 1	Group 2	Group 3	Group 4	Group 5	*P value*
Leukocytes (×10^3^/μL)						
*Me* ± *SD*	5,85 ± 2,12	6,07 ± 1,15	4,96 ± 3,36	4,65 ± 3,24	14,95 ± 14,43	0.762
*Med* [*Q*1; *Q*3]	5,3 [4,6; 7,2]	6,1 [4,9; 7,2]	5,5 [3,2; 7,2]	4,3 [2,9; 8,0]	10,1 [4,7; 25,2]	
*Min*–*Max*	4,0–8,9	4,9–7,2	0,2–8,7	0,1–8,4	4,4–35,3	
Lymphocytes (×10^3^/μL)						
*Me* ± *SD*	5,03 ± 1,85	5,17 ± 1,17	5,45 ± 2,22	4,84 ± 2,11	14,45 ± 14,07	0.804
*Med* [*Q*1; *Q*3]	4,7 [3,9; 6,2]	5,4 [3,9; 6,2]	5,7 [3,7; 7,3]	5,0 [2,8; 6,7]	9,6 [4,6; 24,4]	
*Min*–*Max*	3,2–7,6	3,9–6,2	2,8–7,7	2,6–7,1	4,3–34,4	
Monocytes (×10^3^/μL)						
*Me* ± *SD*	0,25 ± 0,10	0,30 ± 0,00	0,20 ± 0,12	0,20 ± 0,19	0,18 ± 0,15	0.763
*Med* [*Q*1; *Q*3]	0,2 [0,2; 0,3]	0,3 [0,3; 0,3]	0,2 [0,1; 0,3]	0,1 [0,1; 0,4]	0,2 [0,1; 0,3]	
*Min*–*Max*	0,2–0,4	0,3–0,3	0,1–0,3	0,0–0,4	0,0–0,3	
Granulocytes (×10^3^/μL)						
*M*e ± *SD*	0,58 ± 0,29	0,67 ± 0,15	0,45 ± 0,17	0,54 ± 0,34	0,33 ± 0,26	0.521
*Med* [*Q*1; *Q*3]	0,5 [0,4; 0,8]	0,7 [0,5; 0,8]	0,5 [0,3; 0,6]	0,4 [0,3; 0,9]	0,3 [0,1; 0,6]	
*Min*–*Max*	0,4–1,0	0,5–0,8	0,3–0,6	0,2–0,9	0,1–0,6	
Lymphocytes (%)						
*Me* ± *SD*	85,4 ± 3,4	84,4 ± 5,0	88,6 ± 5,0	88,2 ± 3,7	96,4 ± 1,2	**0**.**025**
*Med* [*Q*1; *Q*3]	86 [83; 88]	87 [79; 88]	89 [86; 92]	90 [85; 91]	97 [96; 97]	
*Min*–*Max*	81–89	79–88	82–95	84–92	95–97	
Monocytes (%)						
*Me* ± *SD*	4,7 ± 0,7	4,6 ± 0,5	3,7 ± 1,9	2,9 ± 1,5	1,2 ± 0,4	**0**.**039**
*Med* [*Q*1; *Q*3]	5 [4; 5]	4 [4; 5]	3 [2; 5]	2 [2; 4]	1 [1; 1]	
*Min*–*Max*	4–6	4–5	2–6	1–5	1–2	
Granulocytes (%)						
*Me* ± *SD*	9,9 ± 2,8	11,0 ± 4,5	7,7 ± 3,1	9,0 ± 2,4	2,4 ± 0,8	0.063
*Med* [*Q*1; *Q*3]	9 [8; 12]	9 [8; 16]	8 [6; 10]	9 [7; 11]	2 [2; 3]	
*Min*–*Max*	7–14	8–16	4–12	6–12	2–4	
Erythrocytes (×10^6^/μL)						
*Me* ± *SD*	6,55 ± 2,26	7,42 ± 0,91	5,11 ± 2,69	5,61 ± 2,96	6,23 ± 0,90	0.497
*Med* [*Q*1; *Q*3]	7,0 [5,1; 8,0]	7,0 [6,8; 8,5]	5,9 [5,9; 6,0]	6,2 [5,1; 7,8]	6,1 [5,5; 7,0]	
*Min*–*Max*	3,4–8,8	6,8–8,5	0,4–7,3	0,1–8,3	5,3–7,3	
Hemoglobin (g/dL)						
*Me* ± *SD*	10,7 ± 2,9	11,2 ± 0,3	7,8 ± 4,2	8,9 ± 4,6	10,1 ± 1,2	0.427
*Med* [*Q*1; *Q*3]	11 [9; 13]	11 [11; 11]	9 [9; 10]	10 [9; 12]	10 [9; 11]	
*Min*–*Max*	7–14	11–11	0–11	0–13	9–12	
Hematocrit (%)						
*Me* ± *SD*	28,3 ± 8,7	31,2 ± 3,6	21,9 ± 11,4	28,7 ± 4,8	27,8 ± 3,2	0.428
*Med* [*Q*1; *Q*3]	30 [23; 34]	29 [29; 35]	25 [25; 27]	26 [26; 32]	28 [26; 30]	
*Min*–*Max*	16–37	29–35	2–31	24–35	23–31	
MCV (fL)						
*Me* ± *SD*	43,8 ± 2,5	42,1 ± 0,4	43,3 ± 2,3	43,0 ± 2,3	45,1 ± 6,0	0.804
*Med* [*Q*1; *Q*3]	43 [42; 45]	42 [42; 43]	43 [42; 45]	42 [42; 43]	42 [42; 48]	
*Min*–*Max*	42–48	42–43	41–46	41–47	42–54	
MCH (pg)						
*Me* ± *SD*	16,8 ± 1,8	15,2 ± 1,6	14,2 ± 2,7	16,0 ± 0,7	16,3 ± 1,4	0.335
*Med* [*Q*1; *Q*3]	16 [16; 18]	16 [13; 16]	15 [15; 15]	16 [16; 16]	16 [15; 17]	
*Min*–*Max*	16–20	13–16	10–17	16–17	15–18	
MCHC (g/dL)						
*Me* ± *SD*	38,3 ± 1,9	36,1 ± 3,6	32,9 ± 6,6	37,3 ± 0,7	36,2 ± 1,6	0.129
*Med* [*Q*1; *Q*3]	38 [37; 40]	37 [32; 39]	35 [35; 36]	37 [37; 38]	37 [35; 37]	
*Min*–*Max*	37–41	32–39	21–37	37–38	34–37	
RDW (%)						
*Me* ± *SD*	19,9 ± 1,0	19,6 ± 0,2	20,8 ± 1,9	21,0 ± 2,0	23,6 ± 8,7	0.618
*Med* [*Q*1; *Q*3]	20 [19; 21]	20 [20; 20]	21 [20; 21]	20 [20; 20]	19 [19; 28]	
*Min*–*Max*	19–21	20–20	19–24	20–25	19–37	
PLT (tys./μL)						
*Me* ± *SD*	207 ± 53	305 ± 73	410 ± 201	490 ± 434	652 ± 281	0.072
*Med* [*Q*1; *Q*3]	223 [169; 245]	271 [255; 389]	453 [447; 511]	413 [264; 535]	563 [462; 843]	
*Min*–*Max*	133–249	255–389	63–578	19–1296	429–1054	
MPV (fL)						
*Me* ± *SD*	6,7 ± 1,2	5,9 ± 0,2	6,2 ± 0,4	6,9 ± 1,1	6,1 ± 0,2	0.488
*Med* [*Q*1; *Q*3]	6 [6; 7]	6 [6; 6]	7 [6; 7]	7 [6; 8]	6 [6; 6]	
*Min*–*Max*	6–9	6–6	6–7	6–8	6–6	
PDW (fL)						
*Me* ± *SD*	47,4 ± 7,4	47,8 ± 1,8	34,1 ± 7,9	31,0 ± 12,9	31,6 ± 10,5	0.080
*Med* [*Q*1; *Q*3]	50 [43; 52]	47 [46; 50]	37 [27; 41]	34 [23; 40]	36 [25; 38]	
*Min*–*Max*	37–53	46–50	25–42	10–45	16–38	
PCT (%)						
*Me* ± *SD*	0,133 ± 0,016	0,179 ± 0,041	0,262 ± 0,133	0,311 ± 0,241	0,397 ± 0,169	0.073
*Med* [*Q*1; *Q*3]	0,14 [0,12; 0,15]	0,16 [0,15; 0,23]	0,29 [0,27; 0,34]	0,28 [0,18; 0,39]	0,33 [0,29; 0,51]	
*Min*–*Max*	0,11–0,15	0,15–0,23	0,04–0,38	0,02–0,73	0,28–0,64	

Me – mean; SD – standard deviation; Med – median; Q1 – lower quartile; Q3 – upper quartile; Min – minimal value; Max – maximal value, *p* – significance.

## Discussion

In clinical oncology, anticancer drugs are often used in combination. The discovery of useful combination chemotherapy is expected to increase the response rate and the frequency of long-term survival^18^. The results of our study show that insulin pretreated colon cancer cells are significantly more susceptible to commonly used chemotherapeutics: FU, IRI, OXA, DOC. The apoptosis ratio was also enhanced when insulin was administered together with the examined drugs. The *in vivo* analysis confirmed that insulin could enhance the effect of FU while showing no toxicity. Insulin production is limited to β-cells of the pancreas, and under the normal condition, it is strictly regulated by the concentration of serum glucose. Contrary to the epidermal growth factor and other tissue growth factors that are significant for growth promotion of malignancies, insulin acts as a classic hormone, affecting cells and tissues distant from its site of release. Aberrant autocrine production of insulin by cancer tissues is rare^19^. Insulin binds to the membrane receptors of the insulin-responsive cells, which express high levels of INSR. The insulin receptor is a representative of the tyrosine kinase class of membrane receptors and is homologous to oncogenes of the tyrosine kinase class. The insulin receptors possess the ability to autophosphorylate and transphosphorylate intracellular substrates, and in turn, initiate a cascade of complex cellular reactions. The activated INSR tyrosine kinase initiates several substrates including insulin receptor substrate proteins (IRS1-4), Phosphatidyl Inositol 3-Kinase (PIK3), Akt, MAPK, and signal regulatory protein family^18,20^. Our genetic and protein analysis revealed that cancer cells exposed to drug treatment exhibited significantly lowered mRNA concentration of the key substrates. The results indicate that additional pretreatment with insulin resulted in a significantly lower expression of PIK3CA and GRB2 mRNA and protein when compared with the drug-only treated cells. Genetic research indicates that the PIK3 pathway is the most frequently altered pathway in malignancies, with PIK3CA being the second most frequently mutated oncogene^21,22^. This common oncogenic driver that is central to all malignant cells is recognized as a key target for novel cancer compounds^23^. Upregulation in the PIK3 signaling network provides tumor cells with enhanced capacities for growth, proliferation, survival, and migration. Several ongoing clinical trials in cancer with small-molecule inhibitors against PIK3 report promising results^24^.

**Figure 6:**
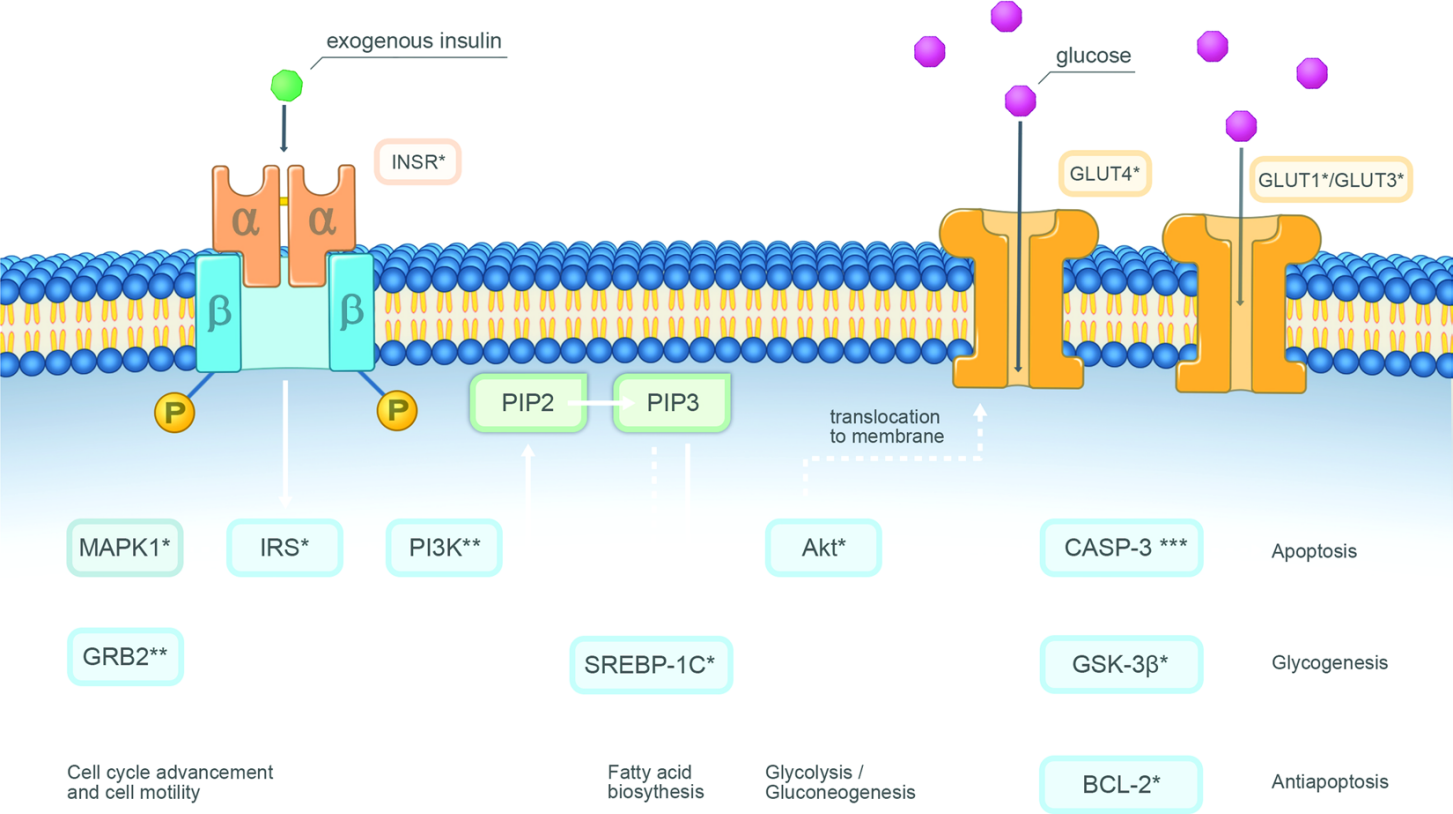
Effects of the therapy on the pathways related to the signaling downstream of insulin receptors. A proposed graphical mechanism illustrates the possible anti-tumor effect of insulin and chemotherapeutic agents in colon cancer cells. Insulin binds to its receptor and initiates a cascade of intracellular processes. Insulin may enhance the therapeutic effect of anticancer drugs through the downregulation of GRB2, a crucial substrate for cell cycle advancement and cell motility, as well as PIK3CA, which plays a pivotal role in growth, proliferation, survival, and migration of cancer cells. *Downregulated after the treatment with cytotoxic agent alone and in combination with insulin. **Downregulated only in insulin pretreated cells.

Research supports the role of insulin and IGF-1 as important growth factors, acting through the tyrosine kinase growth factor cascade in enhancing abnormal tumor growth^25^. Increased insulin levels have been regarded as a crucial factor for the poor prognosis of obesity-associated cancer. Moreover, aberrant insulin and IGF signaling axis have been associated with numerous malignancies including breast cancer, colorectal cancer, prostate cancer, pancreatic cancer, melanoma, osteosarcoma, and childhood malignancies^26^. Constitutively high level of insulin present in the environment initiates a cascade of phosphorylation events leading to activation of several pathways including the PI3K pathway and ultimately results in the highly proliferative and invasive cancer phenotypes^25–27^. However, in our study, insulin over 48 hours in culture did not upregulate the PI3K pathway. The discrepancy in the inhibitory effect of insulin may be due to the temporal pattern of action. During our study, as well as previously reported *in vitro* and clinical studies^9–16^, the concentration of insulin is elevated only for a limited time. The conflicting results regarding the effect of insulin on the PI3K pathway may be explained due to the relatively short duration of action. Moreover, the concentration of insulin used in the *in vitro* experiment was higher than circulating concentrations observed in a clinical setting or concentrations used in the previous studies^9–16^. Higher doses of insulin are known to activate IGF1R-related signaling and affect the propensity for invasion and metastasis. These apparently conflicting results on insulin and IGF1R signaling pathway can result from differences in doses and duration of action of insulin on the cells. High doses of insulin and a short duration of action may account for the effect observed in our experiments. Notably, the *in vitro* findings may not be accountable for a sensitizing effect of acute administration *in vivo*. The results of our study indicate that the downregulation of this key substrate may be responsible for the sensitizing effect of insulin. GRB2 signaling acts as a controlling stage and is crucial for cell cycle advancement and cell motility, and therefore, more intricate processes such as morphogenesis, angiogenesis, and vasculogenesis^28^. Research shows that many types of malignancies, such as breast cancer, are characterized by upregulated GRB2-MAPK pathway, especially since GRB2 is coded in a chromosome region duplicated in many types of neoplasms^15,29,30^. Upregulated GRB2 is also correlated with the likelihood of metastasis in colon cancer^31^. Changes of the effect of the chemotherapeutics on cells can also be explained by the fact that GRB2 is a well-known modifier of cell endocytosis^32^. Stopping GRB2-MAPK pathway at GRB2 point is also a proven way to introduce cells to the apoptotic pathway^33^. Hence, insulin affecting cancer cell metabolism by downregulating the GRB2 may have key meaning for the planning of novel treatments. The combination of insulin with FU/IRI did not alter the expression of other mRNA substrates when compared to the drug only. No significant changes were observed in insulin-only treated cells when compared with control cells. These results implicate that insulin alone does not promote growth and proliferation-related processes in cancer cells. In all cells, the expression of INSR was significantly downregulated. The downregulation of INSR resulted in a lower expression of the insulin receptor substrate 1, which controls a variety of downstream responses, including cell cycle advancement, cell motility, fatty acid biosynthesis, glucose uptake, glycogenesis, glycolysis, gluconeogenesis and antiapoptosis. The pathways that control most hallmarks of cancer were downregulated. The results are presented in Fig. 6. Circulating tumor cells (CTCs) are cancer cells that emerge from solid neoplasms, travel into the blood system, and can form distant metastases. They have been described to be a surrogate biomarker for cancer treatment response in several cancers, i.e., primary breast malignancies^34^, and their appearance has been associated with shorter survival in cases with advanced breast, prostate, colorectal and lung cancer^35^. CTCs may enable more sensitive monitoring of treatment efficacy and thereby guide drug selection^36^. Also in the preclinical setting, the enumeration of CTCs is useful for tracking metastasis, developing biomarkers and testing new drugs^37^. Interestingly, in this study, we found that the addition of insulin to FU resulted in a significantly lowered number of CTCs. The results indicate that the addition of insulin may improve the outcome of systemic chemotherapy by limiting the number of CTCs. The relationship between insulin and cancer has been of interest among scientists and physicians for decades. Conflicting results regarding the relationship between the role of insulin in cancer progression and cancer treatment have been reported. Much of the controversy surrounding this issue may be ascribed to differing definitions and methodologies that make it difficult to draw satisfactory conclusions.

Our data suggest that insulin enhances the effect of chemotherapeutic agents in colorectal cancer while showing no toxicity. The underlying mechanism can be related to the downregulation of PIK3CA and GRB2, which are crucial for growth, proliferation, survival, and migration of cancer cells. Further *in vivo* and clinical experiments are required to unequivocally resolve the issue, which, in turn, may lead to the development of new and inexpensive strategies for the clinical chemotherapy of tumors.

## Methods

### Cell culture and experiment conditions

The human colon adenocarcinoma cell lines Caco-2 and SW480 were purchased from Leibniz Institute DSMZ-German Collection of Microorganisms and Cell Cultures. SW480 cells were maintained in RPMI 1640-GlutaMax supplemented with heat-inactivated 10% fetal bovine serum and 1% penicillin-streptomycin. Caco-2 cells were maintained in 80% MEM- Glutamax supplemented with 20% heat-inactivated fetal bovine serum and 1% penicillin-streptomycin. Cells were incubated at 37 °C in a 5% CO2 and 95% humidified atmosphere to 80% confluence. Cell culture reagents were purchased in Life Technologies (Thermo Fisher Scientific, USA). For all experiments, cells were detached with 0.25% trypsin-EDTA, centrifuged, and seeded for experiments in an appropriate amount. The following cells were exposed to insulin (Insulin solution human, Sigma Aldrich, Merck KGaA, Darmstadt, Germany) for 8 hours in dose 100 µg/ml and then treated for a further 48 hours by tested chemotherapeutics.

### Drugs preparation

5-fluorouracil (FU) and cyclophosphamide (CPA) were purchased in Sigma Aldrich (Merck KGaA, Darmstadt, Germany) and oxaliplatin (OXA), irinotecan (IRI) and docetaxel (DOC) in Selleckchem (Munich, Germany). Aliquots of drugs were prepared according to the manufacturer’s instructions and stored at −80 °C. For each experiment, compounds were freshly diluted to the desired concentrations in the culture medium. Control cells were grown in culture medium or medium with 0,05% DMSO (BioShop Canada Inc., Ontario, Canada) concentrations maximally.

### Cell viability and MTT assay

The viability of Caco-2 and SW480 cells in response to the insulin and chemotherapeutics was determined by the MTT reduction assay (Sigma Aldrich, Germany). Cells were cultured in 96-well culture (7–10 × 10^3^/well) and treated as described above with different chemotherapeutics concentrations for 48 hours. The concentration of a drug that is required for 50% inhibition (IC50) was established during preliminary studies and used in the study. Caco-2 cells were treated with: FU (1) 500 µM, (2) 100 µM; IRI (1) 150 µM, (2) 50 µM; OXA (1) 50 µM, (2) 15 µM; DOC (1) 4000 nM, (2) 1000 nM; CPA (1) 15 µM, (2) 4 µM, while SW480 cells were treated with FU (1) 500 µM, (2) 250 µM; IRI (1) 200 µM, (2) 100 µM; OXA (1) 198 µM, (2) 96 µM; DOC (1) 4000 nM, 92) 100 nM; CPA (1) 12 µM, (2) 6 µM. Freshly prepared MTT solution in culture medium was added to the wells to a final concentration of 0.5 mg/ml and incubated at 37 °C for 4 h. The violet formazan crystals were solubilized with 100 μl DMSO (Sigma Aldrich, Germany) for 15 minutes. The optical absorbance (OA) was measured at 490 nm by a BioTek ELX800 multiwell reader (BioTek, Winooski, VT, USA). The 100% viability of cells was determined as absorbance of the untreated control groups, and treated groups were calculated according to the formula:

$${\rm{Viable}}\,{\rm{cells}}\,( \% )=({\rm{OA}}\,{\rm{of}}\,{\rm{experimental}}\,\mathrm{group}/\mathrm{OA}\,{\rm{of}}\,{\rm{control}}\,{\rm{group}})\times 100.$$

For further experiments, FU and IRI were used in a concentration of 500 µM and IRI 50 µM, respectively. The dose of insulin was specified to determine the level that did not cause any significant effect in cell viability according to the control group. All assays were repeated 3 times.

### Flow cytometry analysis

Caco-2 and SW480 apoptotic cells ratio following fluorouracil (500 µM) and irinotecan (50 µM) alone and with 8 h insulin pretreatment were measured using Muse® Annexin V and Dead Cell Assay Kit (Merck KGaA, Darmstadt, Germany). Cells were seeded in 6-well culture plates (1,5 × 10^5^/well) and treated as described in experimental conditions. First, cells were washed with PBS, incubated with 100 ul/well Gibco™ Trypsin-EDTA, and after 5 minutes, 300 µl of culture medium was added to each well. Next, cells were transferred to Eppendorf® tubes and gently mixed on vortex. 100 μl of each cell sample and 100 µl of Muse™ Annexin V & Dead Cell reagent were incubated for 20 minutes at room temperature in the dark. Samples were analyzed by Muse™ Cell Analyzer (Merck KGaA, Darmstadt, Germany), where the apoptotic cells were examined. The results include the percentage of live, early apoptotic, late apoptotic, total apoptotic, and dead cells in dot plots showing the binding of Annexin V-FITC and PI uptake.

### RNA isolation and quantitative real-time PCR

Total RNA was isolated from cell lines, including DNase treatment using an RNeasy Mini Kit and RNase-Free DNase Set from Qiagen (Hilden, Germany) according to the manufacturer’s instructions. The concentration of mRNA was measured by the Qubit RNA BR Assay for the Qubit Fluorometer (Invitrogen, Thermo Fisher Scientific Inc., Waltham, Massachusetts, USA). 2 µg of RNA samples were subsequently reverse transcribed to generate complementary cDNA with Qiagen RT2 First Strand Kit using MJ Research PTC-100 PCR Programmable Thermal Controller. Quantitative reverse transcription–polymerase chain reaction (qRT-PCR) was performed with an RT^2^ SYBR Green qPCR Mastermix and Custom RT2 Profiler PCR Arrays from Qiagen (Hilden, Germany), using Light Cycler 480 96-well block (Basel, Switzerland). The template includes eight controls: 5 Housekeeping Genes (ACTB, HPRT1, TBP, B2M, GAPDH), 1 Genomic DNA Control (GDC), 1 Reverse Transcription Control (RTC) and 1 PCR Positive Control (PPC). Ct values were analyzed in the Qiagen web portal at GeneGlobe. Ct values were normalized based on reference genes and fold change using the ΔΔCt method, in which ΔCt is calculated between the gene of interest (GOI) and an average of housekeeping genes (HKG). Fold Change is then calculated using 2^−ΔΔCt^ formula.

### Western blotting analysis

Caco-2 and SW480 cells were seeded in 6-well culture plates (9 × 10^4^/well). The cells were washed with PBS, then the appropriate amount of RIPA buffer containing 1% protease and phosphatase inhibitors (Sigma Aldrich, Germany) was added to each well. The cells were scraped and transferred to Eppendor® tubes for low agitation for 30 min at 4 °C and then centrifuged at 16000 × g for 20 minutes. The protein level was measured at 280 nm using the Qubit Protein Assay for the Qubit Fluorometer (Invitrogen, Thermo Fisher Scientific Inc., Carlsbad, CA, USA). 50 ug protein extracts were separated on NuPAGE gels 4–12% in NuPage MES SDS Running Buffer. After electrophoresis, proteins were transferred to the nitrocellulose membrane in NuPAGE Transfer Buffer (Invitrogen, Thermo Fischer Scientific, USA). Then the nitrocellulose membrane was blocked with 10% goat serum (Sigma Aldrich, Germany) in PBST for 1 hour at room temperature and incubated overnight at 4 °C with the first antibodies: polyclonal anti-β-actin for protein normalization (dilution 1:1000, Abcam, Cambridge, UK), polyclonal anti-PI3K (dilution 1:100, Boster, USA), polyclonal anti-GRB2 (dilution 1:100, Sigma Aldrich, Germany). The next day, the nitrocellulose membrane was washed three times for 10 minutes in PBST, incubated with a secondary anti-rabbit antibody in dilution 1:1000 (Santa Cruz Biotechnology Inc., Santa Cruz, CA, USA) labeled with horseradish peroxidase for 1 h at room temperature. After washing 3 × 10 min in PBST, protein bands were visualized by the 1-Step Ultra TMB Blotting Solution (Thermo Fisher Scientific, USA). The membrane documentation was performed by Molecular Imager Gel Doc TMXR+ (BioRad, Hercules, CA, USA). Unprocessed original scans for the blots presented in Figure 3 are presented in the supplementary information. 

### Mouse allograft model of colon cancer

C57BL/6 female, 12–16-week-old mice, weighing 20–25 g were obtained from the Medical University of Bialystok (Bialystok, Poland) and maintained under specific pathogen-free (SPF) conditions. All experiments were performed according to the EU Directive 2010/63/EU for animal experiments and were approved by the 1^st^ Local Committee for Experiments with the Use of Laboratory Animals, Wroclaw, Poland (Permission No. 51/2018 issued on 16/05/2018). The mouse colon adenocarcinoma MC38 cells were obtained from Tumor Bank of Radiobiology Institute TNO, Rijswijk, Holland, and established for *in vitro* culture at the Institute of Immunology and Experimental Therapy. MC38 cells were cultured *in vitro* in RPMI-1640 medium (IIET, Wroclaw, Poland) supplemented with 1 mg/mL geneticin (Gibco, UK), 2 mM L-glutamine, 1 mM sodium pyruvate (both from Sigma-Aldrich, Germany), 5% fetal bovine serum (HyClone, Thermo Fisher Scientific Inc., UK). Cells were trypsinized (IIET, Poland), centrifuged (200 g, 4 °C, 5 min $TV=\frac{1}{2}\times {a}^{2}\times b\,{\rm{\min }}$), and counted prior to transplantation. Subcutaneous transplantation: mice were subcutaneously (s.c.) inoculated in the right flank region with 1 × 10^6^ cells suspended in 0.2 mL saline per mouse. After the tumor inoculation (day 0), 50 mice were randomly divided into 5 different groups (10 per group). All animals were monitored for activity, physical condition, body weight, and tumor growth. Tumor size was determined every other day by caliper measurement of two perpendicular diameters of the implant. Tumor volume (TV, in mm^3^) was calculated with the formula: in which a is the long diameter and b is the short diameter (in mm). Mice with a tumor over 2000 mm^3^ and locomotor disorders or poor condition diagnosed by a veterinary physician were euthanized. Mice lost during the experiment: 2 in group 1; 2 in group 2; 1 in group 3; 1 in group 4; 2 in group 5.

### *In vivo* chemotherapy

The animals bearing tumors were randomly divided into five groups (5–10 mice per group): 1- control, 2- insulin only, 3- FU only, 4- insulin with FU administered together, 5- insulin administered 60 minutes prior to FU. The untreated control group received the vehicle only. The dose of insulin was established during preliminary studies. Insulin was administered s.c. at a dose of 2.5 U/kg/d 1 d/wk for 3 wks. FU was dissolved in the vehicle, 0.9% saline, and given intraperitoneally (i.p.) at doses of 100 mg/kg/d, 1 d/wk for 1 wk; 150 mg/kg/d, 1 d/wk for 1 wk and 100 mg/kg/d, 1 d/wk for 1 wk. The insulin was administered either together with FU or 60 minutes prior to the injection. At the end of the experiments, tumors, pancreas, and livers were removed and processed to formalin-fixed and paraffin-embedded (FFPE) blocks for histological analysis. The blood from mice was collected to ethylenediaminetetraacetic acid (EDTA) tubes and used separately for the analysis of complete blood count and circulating epithelial tumor cells.

### Complete blood count

Blood samples (500 µl–1000 µl) were drawn into normal blood count tubes with ethylenediaminetetraacetic acid (EDTA) as an anticoagulant and processed within 48 hours of collection. The level of leukocytes, lymphocytes, monocytes, granulocytes, erythrocytes, hemoglobin, hematocrit, MCV, MCH, MCHC, RDW, platelets, MPV, PDW, PCT, was measured in each blood sample (Sysmex K4500SL, serial number F2872, Japan).

### Circulating tumor cells (CTCs)

Circulating tumor cells (CTC) were successfully enumerated from peripheral blood using maintrac® approach, as reported previously^38^. In short, 500 ul blood was incubated with lysis buffer (Qiagen, Hilden, Germany) to remove red blood cells, and all nucleated cells were resuspended in 500 ul PBS-EDTA. Subsequently, CTC were stained with 4 µl fluorescently labeled antibodies directed against EpCAM (clone caa7-9G8, Miltenyi Biotec GmbH, Germany) and incubated for 15 min in the cold. Then, the samples were diluted with PBS-EDTA, and a defined volume of the cell suspension and propidium iodide (PI) (Sigma-Aldrich, USA) was transferred to 96 well plates (Greiner Bio-one, USA). Finally, we imaged and enumerated CTC using a fluorescence scanning microscopy (ScanR, Olympus, Tokyo, Japan), enabling visual examination of vital tumor cells. Vital CTCs were defined as EpCAM-positive cells, lacking in nuclear PI staining and with intact morphology, and only these cells were counted. Quality control regarding reagents, instrument standardization, and operator technique was assessed.

### Histological evaluation of toxicity

Formalin-fixed and paraffin-embedded tissue sections of tumors, livers, and pancreas were stained with hematoxylin-eosin (HE) to evaluate the impact of the insulin and chemotherapeutics on the tissues.

### Statistical analysis

The conformity of the distribution of analyzed parameters with the normal distribution was examined. The conformity was evaluated by the Shapiro-Wilk test. The homogeneity of the variance was tested with Bartlett’s test. The significance of differences in mean values (Me) in 5 groups, for parameters with a non-normal distribution, was checked using the nonparametric Kruskal-Wallis test. In case of rejection of the null hypothesis with equality of average values in groups (median), to verify the differences between average values in pairs posthoc tests (Dunn test multiple comparisons) were carried out. The significance of differences in mean values (Me) in more than two populations for parameters of normal distribution and homogeneous variances was evaluated with analysis of variance (ANOVA). In case of rejection of the null hypothesis of homogeneity of variance, to verify the differences between the mean values in pairs, posthoc tests were conducted (Scheffe’s test). The level p = 0.05 was considered as the critical significance level. Data were represented as mean ± standard deviation (Me ± SD). The analysis of the obtained results was carried out using the STATISTICA v.13 program (StatSoft, Inc. Tulsa, OK. the USA).

## Study Highlights

New anticancer drugs that marginally improve survival of patients continue to be developed at an unsustainably high cost. The study aimed to elucidate the effects of insulin, an inexpensive drug with a convincing safety profile, on the susceptibility of colon cancer to commonly used chemotherapeutic agents. The *in vitro* and *in vivo* study showed that insulin enhanced the antitumor effect of chemotherapeutics via downregulation of PIK3CA and GRB2, that play a critical role in cell signaling. Insulin might be potentially applied to clinical use to enhance the therapeutic effectiveness of chemotherapeutic drugs. The findings may become a platform for future development of new and inexpensive strategies for the clinical chemotherapy of tumors.

## Supplementary information

Supplementary Information File
