# Supplementary material for: Insulin enhancement of the antitumor activity of chemotherapeutic agents in colorectal cancer is linked with downregulating PIK3CA and GRB2
Source: Sci Rep. 2019 Nov 12;9:16647. doi: 10.1038/s41598-019-53145-x (PMC6851401; doi:10.1038/s41598-019-53145-x)
Supplement: Supplementary file 1 — Supplementary Information File [file 41598_2019_53145_MOESM1_ESM.pdf]

Supplementary information file

Insulin enhancement of the antitumor activity of chemotherapeutic agents in colorectal cancer is linked with downregulating PIK3CA and GRB2

Siddarth Agrawal[1], Marta Woźniak[1], Mateusz Luc[1], Sebastian Makuch[1], Ewa Pielka[1], Anil Kumar Agrawal[2], Joanna Wietrzyk[3], Joanna Banach[3], Andrzej Gamian[4], Monika Pizon[5], Piotr Ziółkowski[1]

1. *Department of Pathology, Wroclaw Medical University, Wroclaw, Poland*
2. *2nd Department and Clinic of General and Oncological Surgery, Wroclaw Medical University*
3. *Department of Experimental Oncology, Ludwik Hirszfeld Institute of Immunology and Experimental Therapy, Polish Academy of Sciences*
4. *Department of Biochemistry, Wroclaw Medical University*
5. *Transfusion Center Bayreuth, Kurpromenade 2, 95448 Bayreuth, Germany*

Corresponding author

Siddarth Agrawal

Department of Pathology, Wroclaw Medial University

Marcinkowskiego St.1, 50-368 Wroclaw, Poland

Tel.: +48 71 784 12 12, Fax: +48 71 784 00 57

siddarth@agrawal.pl

All authors declare that they have no competing interests.

Research was funded by the Wroclaw Medical University grant no.: ST.A010.17.006 and grant no.: STM.A011.17.055 and Polish National Science Center (NCN) grant no. 2015/19/N/NZ5/00001

Keywords: insulin; chemotherapy; colorectal cancer; fluorouracil

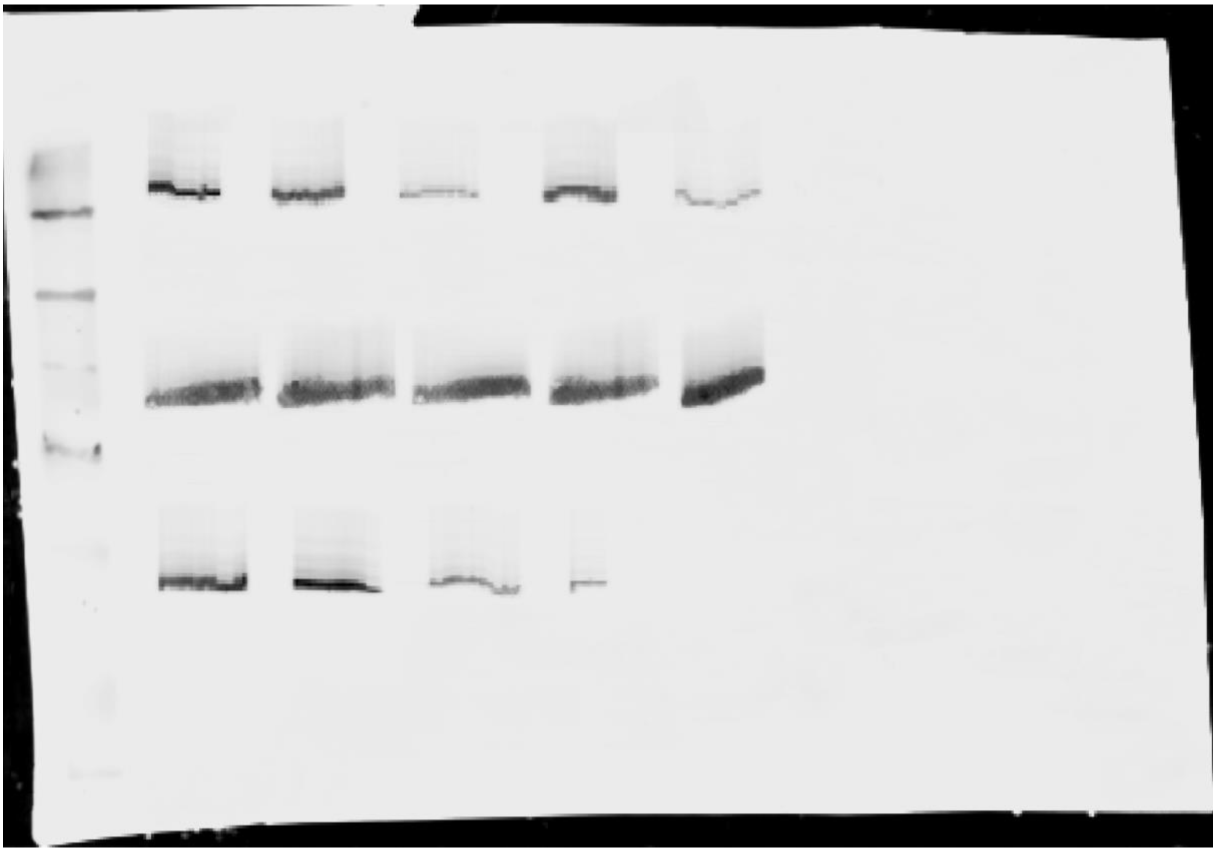

Supplementary Figure S1. Unprocessed original scans for the blots presented in Figure 3 – Caco-2 cell line. The bands are (starting from the top): PI3K p85 (85kDa); Beta actin (44kDa); GRB2 (25 kDa). Bands represent (starting from the left): control, irinotecan, irinotecan + insulin; fluorouracil; fluorouracil + insulin.

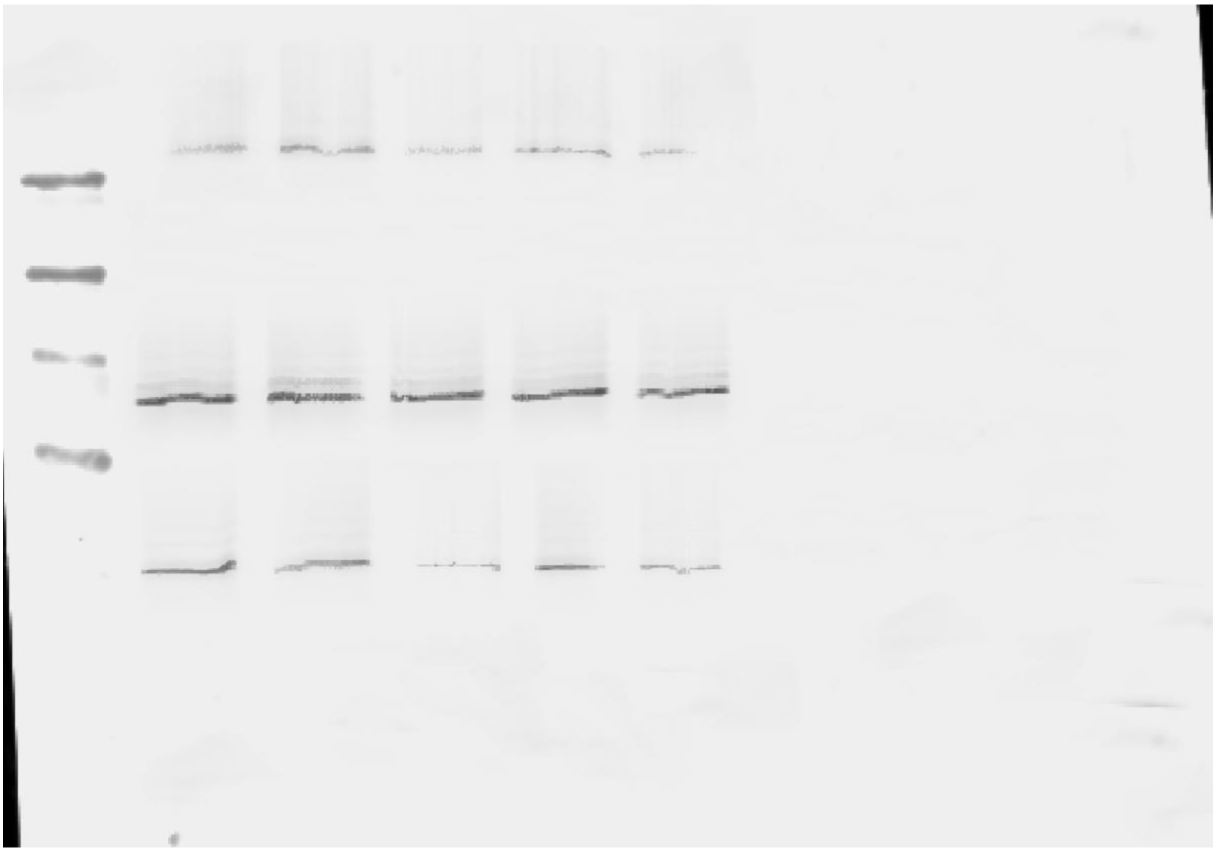

Supplementary Figure S2. Unprocessed original scans for the blots presented in Figure 3 – SW480 cell line. The bands are (starting from the top): PI3K p85 (85kDa); Beta actin (44kDa); GRB2 (25 kDa). Bands represent (starting from the left): control, irinotecan, irinotecan + insulin; fluorouracil; fluorouracil + insulin.
